# What can measures of text comprehension tell us about creative text production?

**DOI:** 10.1007/s11145-015-9551-6

**Published:** 2015-02-15

**Authors:** Lisanne T. Bos, Björn B. de Koning, Floryt van Wesel, A. Marije Boonstra, Menno van der Schoot

**Affiliations:** 1 Department of Educational Neuroscience and LEARN! Research Institute for Learning and Education, Faculty of Psychology and Education, VU University Amsterdam, van der Boechorststraat 1, 1081 BT Amsterdam, The Netherlands; 2 Unit Contract Research, Department of Research and Innovation, CED-Groep. Educative Services, Dwerggras 30, 3068 PC Rotterdam, The Netherlands

**Keywords:** Creative writing, Text comprehension, Situation-model, Path model

## Abstract

Evidence is accumulating that the level of text comprehension is dependent on the situatedness and sensory richness of a child’s mental representation formed during reading. This study investigated whether these factors involved in text comprehension also serve a functional role in writing a narrative. Direct influences of situatedness and sensory richness as well as indirect influences via the number of sensory and situational words on the creativity (i.e., originality/novelty) of a written narrative were examined in 165 primary school children through path analyses. Results showed that sensory richness and situatedness explained 35 % of the variance in creativity scores. Sensory richness influenced the originality/novelty of children’s narrative writing directly, whereas situatedness had an indirect influence, through the number of sensory words, but both pathways influenced the outcomes to a comparable extent. Findings suggest that creative writing requires similar representational processes as reading comprehension, which may contribute to the development of instructional methods to help children in creative writing assignments.

## Introduction

Comprehending written text and writing comprehensible text are important skills that children are required to master in primary education (Broekkamp, Janssen, & van den Bergh, 2009; Choo, 2010; Lancia, 1997; Olson & Oatley, 2013). Reading comprehension and writing traditionally have been considered to be related activities involving related language processes (Langer & Fliban, 2000). Evidently, readers and writers both work towards constructing meaning (Choo, 2010; Tierney & Pearson, 1983) and engage in meaning-making activities that require similar prior knowledge and experiences with the topic, knowledge about language, knowledge about structure and so on (e.g., Kucer, 1987). However, text comprehension and text production mainly constitute distinct bodies of research (Langer & Fliban, 2000). The present study aims to bring together these two related, yet largely unconnected, areas of research. The focus will be on the extent to which two components of meaning-making drawn from reading comprehension research (i.e., situatedness and sensory richness) also contribute to primary school children’s creative writing, which was operationalized as the originality/novelty of a produced narrative (e.g., Barbot et al., 2012; Sternberg & Lubart, 1999).

### Text comprehension

In recent years, there has been substantial interest in reading comprehension and the factors associated with becoming a proficient reader (Glenberg, Brown, & Levin, 2007; Kendeou, Smith, & O’Brien, 2013; Yuill & Oakhill, 1988; Zwaan & Radvansky, 1998). According to contemporary theories of reading comprehension, such as the situation-model framework (Zwaan & Radvansky, 1998), comprehending text involves constructing a mental representation of the described situation. This so-called situation-model representation is gradually build up along a number of key situational dimensions such as the story’s protagonist, time, space, causality and intentionality (Zwaan, Langston, & Graesser, 1995). By integrating this situational information with the readers’ background knowledge, a non-linguistic, coherent, and integrated mental representation of the ‘state of affairs’ described in a text is formed (for more detailed information, see Zwaan & Radvansky, 1998; for an extention to non-narrative text, see Bråten, Britt, Strømsø, & Rouet, 2011). Engaging in these higher-order cognitive processes helps readers to develop an in-depth understanding of a text (Van Dijk & Kintsch, 1983). This way, readers develop a deeper understanding of a text than when they just process words, phrases, and clauses in the text and the relations between them, which results in lower levels of text representation that are linguistic in nature (i.e., text-base or surface-level representations).

Furthermore, the view that situation-model representations formed during language comprehension involve sensory, motor, and emotional information is gaining popularity (e.g. De Koning & van der Schoot, 2013). This view has now been supported by numerous behavioral and neuroimaging studies (for reviews, see Barsalou, 2008; Pulvermüller, 2005). Particularly, according to embodied theories of cognition, readers construct a mental simulation of events described in the text (Kintsch, 1988; van den Broek, 2010). This involves the re-activation of sensory, motor, and emotional experiences which are stored in brain areas responsible for actual perception, action, and emotion, and which the reader has acquired during previous real-world interactions (Barsalou, 2008; Zwaan & Radvansky, 1998). For example, understanding a sentence like ‘She saw the egg in the skillet’ requires the re-activation of perceptual information to simulate the form of the object (egg sunny-side up) which is implied in the sentence (Engelen, Bouwmeester, de Bruin, & Zwaan, 2011; Zwaan & Pecher, 2012). Accordingly, text comprehension requires readers to draw upon the real-world experiences that are stored in all sensory modalities in the brain in order to ‘see’, ‘hear’, ‘feel’, ‘smell’, and ‘touch’ the situations and events described in the text, and in which they can ‘move along’ with the protagonist (Zimmerman & Hutchins, 2003). The idea of reading as a ‘multisensory experience’ to gain an in-depth understanding of the described events is increasingly being acknowledged by reading comprehension researchers (e.g., De Koning & van der Schoot, 2013).

### Text production

So, the extent to which situations and events described in a text are accurately represented depends largely on the level of situatedness and sensory richness of the situation-model representation. A relatively fair amount of research is currently available indicating that these two factors are essential for *comprehension* of narrative text (i.e., from text to situation-model) (Zwaan & Pecher, 2012; Zwaan, Stanfield, & Yaxley, 2002). However, it has not yet been studied whether the situatedness and sensory richness of mental representations can also serve a functional role in *production* of creative narrative text (i.e., from situation-model to text). This is unfortunate, since it can be argued that writers first have to produce a mental representation of the characters, situations, and events of the story they have in mind before starting to write a text (e.g., Bereiter & Scardamalia, 1987; Plum, 1982; for a similar view, see Oatley & Olson, 2010). By determining the situational dimensions like the protagonist, thespatio-temporal setting in which the protagonist acts and the goals he or she seeks to achieve (situatedness), as well as perceptual, action-related, and emotional aspects of the story (sensory richness), writers have access to structural, thematic, and plot-related ‘mental guidelines’ which can help in writing a narrative.

Notably, this argumentation comes close to the main features of the cognitive theory of writing (Flower & Hayes, 1981, 1984; Olson & Oatley, 2013), which states that writing consists of three major processes: planning the text, translating ideas into textual output, and reviewing (i.e., evaluating and revising) the narrative draft as it is written. A similar proposal has been formulated by other cognitive process models of writing such as Bereiter and Scardamalia’s (1987) knowledge transforming model of writing and the ‘simple view of writing’ (Berninger et al., 2002). The processes of writing are presumed to take place in a cyclic, or recursive, sequence rather than following a linear path (Flower & Hayes, 1984; Olson & Oatley, 2013). For example, whereas writing usually starts with planning, according to Murray (1990) people sometimes just start writing without knowing where the story will go, which will become clear along the way during writing. Importantly, given this cyclic approach to writing, for writing to occur successfully writers are required to effectively apply self-regulatory strategies (Zimmerman, 1997). Self-regulatory strategies, such as mental imagery, help people to continuously decide on which information to present, revise or remove as well as where, when and how to describe this information in order to create a meaningful story (Bereiter & Scardamalia, 1987; Zimmerman, 1997).

Especially planning of writing is relevant here in that planning involves generating information to be included in the text, setting goals for the narrative, and organizing information which becomes available from memory (Rogers & Graham, 2008). However, encouraging writers to engage in these cognitive processes is often not sufficient; they need to be provided more direct guidance to do so effectively (Graham & Perin, 2007). Using mental imagery to envision the plot, characters, or setting, like skilled readers do, provides a way to help to accurately describe details or make a story as vivid as possible (Bereiter & Scardamalia, 1987). In our study, this is specifically looked at in terms of embodied situation-model construction. In other words, planning a narrative comes down to creating multisensory images in the mind while actively drawing from one’s own experiences (Barbot et al., 2013). Consistent with this, there is empirical evidence that situation-model representations are needed to, for example, retell a story or identify a theme in it (van den Broek et al., 2005). Moreover, it appears that constructing and recalling visual mental representations enhances writing descriptions or idea generation in creative writing work (Barbot et al., 2013; Flower & Hayes, 1984). Particularly skilled writers appear to naturally use mental imagery while planning and composing text (Bereiter & Scardamalia, 1987). Together, findings suggest a possible role for the situation-model representation, including the situational and sensory elements contained in it, in the text production process.

The present study, therefore, was aimed at investigating whether *reading comprehension* measures tapping the situatedness and sensory richness of mental representations of narrative text also underlie individuals’ creative *writing* of a narrative. Although there is no general consensus on the definition of creative writing (Carlson, 1965; Barbot et al., 2012), most researchers operationalize the concept in terms of novelty and originality (e.g., Barbot et al., 2012; Sternberg & Lubart, 1999; Broekkamp et al., 2009; Nettle, 2009). In this study, narratives are considered creative when they are novel, original, inventive, and unexpected in nature (Sternberg & Lubart, 1999; Carlson, 1965). To help elicit this type of writing, we used an open ended writing assignment (i.e., ‘Write a story beginning with *If I was invisible for one day*….’) which fully enabled children to draw on their creative potential (see also Chen & Zhou, 2010), either with regard to the amount and nature of situational descriptions (i.e., the ‘who’, ‘what’, ‘where’, ‘when’, and ‘how/why’ of story passages) or with regard to the amount and nature of sensory descriptions (i.e., sensory details of sights, sounds, tastes, smells and feelings/textures).

There is, however, limited empirical guidance on the precise interconnections between, situatedness, sensory richness, and creative writing outcome. The few studies that exist on this topic come from creative writing literature (see Zimmerman, 1997), and seem to converge on the idea that creativity of compositions increases when students receive instructions to make (more) use of their senses in their writing (Barbot et al., 2012, 2013; Jampole, Konopak, Readence, Moser, 1991, Jampole, Mathews, Konopak, 1994; Long & Hiebert, 1985). Barbot et al. (2012) investigated creativity of writing in a population of regular children who just entered school (Grades 1 and 2). Children’s perceptual skills were stimulated by teaching them to derive meaning from visual images through visual-literacy practice (i.e., creating a visual mental image of a previously seen illustration). Observations of children’s verbalizations while working in groups and an examination of their story writing skills showed that visual-literacy practice influenced children’s originality of story writing. In a similar vein, Long and Hiebert (1985) found that gifted children in Grade three to six improved in writing quantity and quality after a visual imagery intervention. Jampole et al. (1991) extended previous findings by involving all five senses in their mental imagery training, rather than exclusively focusing on visualization. In their study, 38 gifted children from fourth and fifth grade were randomly assigned to two groups: an imagery training group, who practiced making multi-sensory mental images based on text passages, and a control group who did not receive any training. Results showed that mental imagery training enhanced the creative writing product as indicated by the Carlson Analytic Scale for Measuring the Originality of Children’s Stories (Carlson, 1965). Moreover, the written text also included more sensory words (i.e., visual, auditory, tactile, kinesthetic, olfactory, organic, and gustatory) in the imagery than in the control group. In a subsequent study, Jampole et al. (1994) replicated these findings in a group of third and fourth grade gifted children. Interestingly, mental imagery instructions have not consistently led to similar beneficial effects on creative writing at secondary school level (e.g., Chevreau & Smith, 1989).

### The present study

Together, the above studies suggest a positive relation between mental imagery and creative writing outcomes for primary school children. However, evidence has come almost entirely from the effects of mental imagery instruction in gifted children. To the best of our knowledge, no study yet has examined the extent to which primary school children’s ‘natural’ mental representational abilities to vividly imagine a written story are also related to creative text production. Furthermore, earlier research has typically focused on the sensory dimension of writers’ mental representations while overlooking the situational dimension. Therefore, in this study, we aimed at simultaneously investigating both sensory and situational dimensions enabling us to examine the individual contributions of these two dimensions to the originality/novelty of a written narrative. As we were interested in children’s reliance on their sensory and situational representational skills in naturally writing a narrative, no specific sensory and/or situational instructions were provided. To get insight into these aspects in a broader group of children, we shifted focus from gifted children to normally developing, healthy children with an average level of intelligence. In doing so, there were two questions we specifically wanted to address. First, what are the relative influences of situatedness and sensory richness on creative writing outcome? Second, are these influences direct or indirect? The latter question refers to whether *text comprehension* measures of situatedness and sensory richness (from text to mental representation) can directly predict the assessed originality/novelty of children’s narrative writing, or whether the relationship is mediated by corresponding *text production* measures of situatedness and sensory richness (from mental representation to text), respectively, the actual number of situational and sensory descriptions given in the narrative text they have written.

We took text comprehension measures of situatedness and sensory richness from contemporary theoretical and empirical work on reading comprehension (e.g., De Koning & van der Schoot, 2013; Mcnamara, Magliano, & Mcnamara, 2009). Situatedness was measured using a standardized reading comprehension test (CITO) which provides an indication of the extent to which a created mental text representation is situation-based (Feenstra, Kamphuis, Kleintjes, & Krom, 2010). The extent to which children’s mental text representations involve sensory information (i.e., sensory richness) was measured using the sentence-picture verification task (SPVT; e.g., Zwaan et al., 2002). This task was specifically developed to test whether language comprehension involves use of perceptual, motor, and emotional symbols during text comprehension (Zwaan & Pecher, 2012). To verify our assumption that high scores on these text *comprehension* measures of situatedness and sensory richness would contribute to high scores on the corresponding text *production* measures, we counted the number of situational and sensory characteristics of the narrative texts produced by children. At this, situational descriptions were defined as descriptions along the key situational dimensions of protagonists (‘who’), time (‘when’), space (‘where’), causation (cause-effect connections between text events) and intentionality (character goals). Sensory descriptions, on the other hand, describe what the protagonist sees, hears, smells, touches, and tastes (Holliway, 2004; Schunk & Swartz, 1993) and provide an indication of the liveliness and sensory detail of images in the writer’s mind. Creativity of the written narrative was measured with the ‘Novel Qualities’-subscale of the Carlson Analytic Scale for Measuring the Originality of Children’s Stories (Carlson, 1965), assessing those aspects of text which can be regarded as novel, original, inventive, and unexpected.

**Fig. 1:**
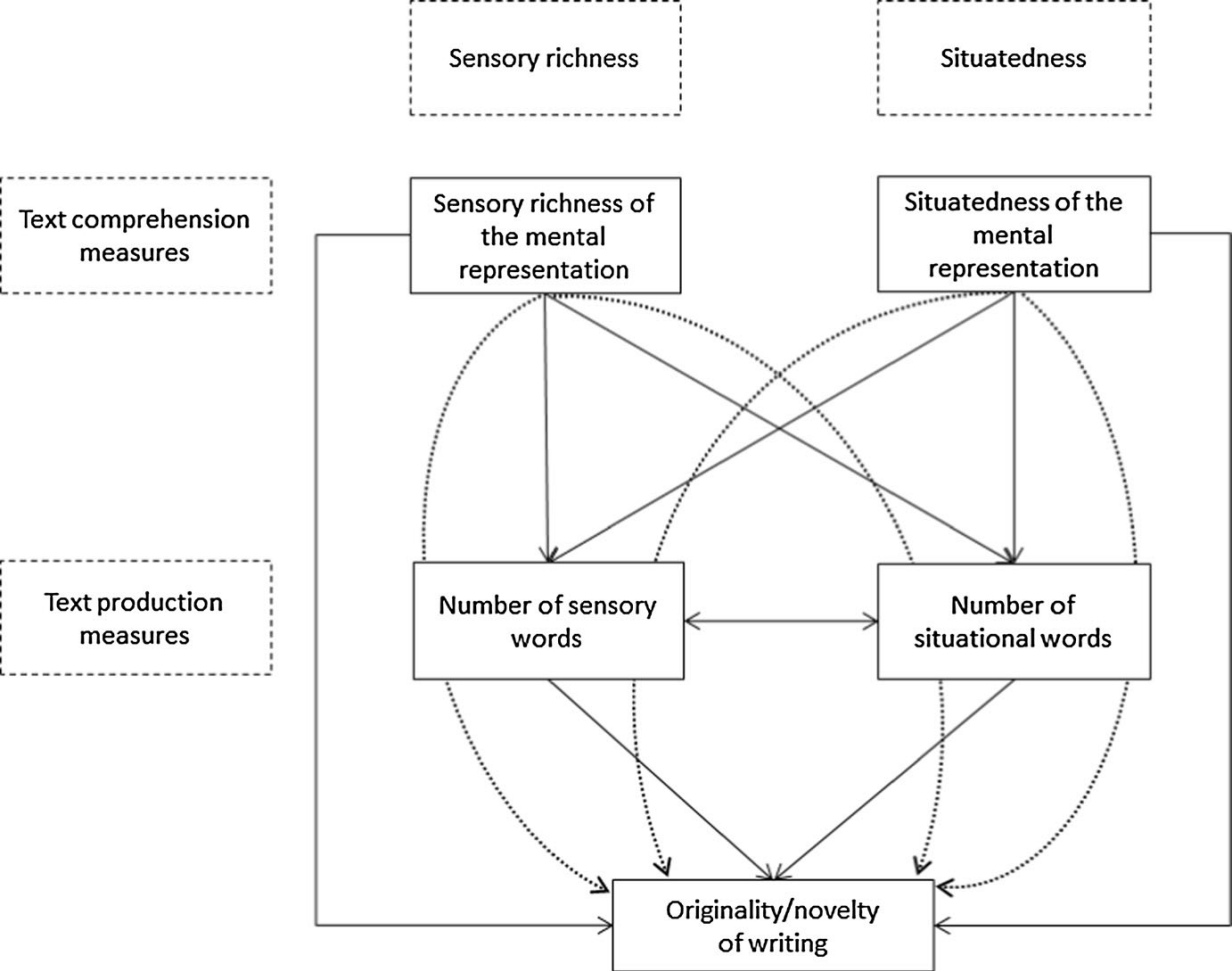
Path model with all hypothesized pathways. *Dashed lines* represent the indirect effects

By using path model analysis, we investigated whether our text comprehension measures of situatedness and sensory richness have a direct effect on the originality/novelty of writing a narrative, or whether these relations are indirect, running via—that is, being completely mediated by—the text production measures of situatedness and sensory richness. In addition, we compared the direct and indirect models against the complete model, in which effects of the two text comprehension measures are partially mediated by their corresponding text production counterparts (see Fig. 1). Using this approach of model comparison allowed us to settle down on the best path model in terms of model fit and model complexity. Although some relations in the path models have been investigated in prior studies, the present study is unique in that it considers all mentioned variables simultaneously within a single study.

## Methods

### Participants

One hundred sixty five children from fourth (*N* = 59), fifth (*N* = 56), and sixth (*N* = 50) grade participated in this study. This sample consisted of 93 boys, (*M*_*age*_ = 10.61 year, *SD*_*age*_ = 0.98 year) and 72 girls (*M*_*age*_ = 10.69 year, *SD*_*age*_ = 0.92 year) from seven public elementary schools in the Netherlands. The schools participated voluntarily. A passive consent was sent out to children’s parents before the start of the study to provide them with information about the study and to offer them the opportunity to withhold their child from participating in this study.

### Materials

#### Writing assignment

To measure the originality/novelty and text production variables, children were asked to write a narrative during their regular writing course. Written narratives were analyzed by a team of six research assistants all of whom were trained prior to rating. They counted the number of sensory words, the number of situational words, and assessed the originality/novelty of the story. To ensure that similar standards of scoring were used by all raters, they used a standardized protocol. In addition, ten randomly selected narratives across the three different grades were scored by all raters to help them reach consensus on the scoring method. Hereafter, all narratives were randomly distributed across the six raters, with each text being scored by two raters. A mean score (based on the two ratings) was calculated for the number of sensory words, the number of situational words, and the assessed originality/novelty.

#### Predictors

##### The sensory richness of the mental representation

To measure the sensory richness of the mental representation, we used the Sentence-Picture Verification Task (SPVT; Zwaan et al., 2002). For this task, children had to read a sentence at their own pace and were subsequently presented with a picture. Then the child had to indicate whether or not the object depicted in the picture was mentioned in the preceding sentence (see Table 1). The task consisted of 72 pictures to accompany 72 sentences divided over 24 filler items and 48 experimental items. The pictures were colored drawings of approximately 15 × 15 cm presented on the computer screen.

**Table 1:** Sample sentences and pictures of the match and mismatch conditions in the sentence-picture verification task

	Match	Mismatch
The chef saw the egg in the fridge		
The chef saw the egg in the skillet		

For the experimental items, each sentence implicitly described a distinct shape of an object. The subsequently presented pictures either matched or mismatched the shape of the object implied in the sentence. For example, the sentence ‘The chef saw the egg in the fridge’ or the sentence ‘The chef saw the egg in the skillet’ was either followed by a picture of an egg in its shell or by a picture of an egg sunny-side up (see Table 1). By crossing the two versions of experimental sentences and the two versions of pictures, four experimental lists were created. Across the four versions, all item combinations were used equally often. On each list, half of the experimental sentence-picture pairs matched whereas the other half mismatched in object shape. As an answer to experimental items always required a yes-response, an equal number of filler items (requiring a no-response) was added to balance responses. Filler items contained sentences of the same form as the experimental items except that they were followed by a picture depicting an unrelated object (i.e., an object not mentioned in the sentence). Children were told that they had to respond as fast and accurate as possible on all sentence-picture trials.

Consistent with previous research using the SPVT, reaction time data were preprocessed using the following procedure (e.g., Zwaan & Pecher, 2012). First, data of two items were removed from the experiment because of low accuracy scores (accuracy below .55) due to the pictures being difficult to recognize. Second, data of two participants were excluded from the dataset. One participant only gave no-responses on all trials, whereas another participant gave no-responses on all corresponding pictures in the mismatch condition, reflecting inaccurate understanding of the task. Third, reaction times shorter than 300 ms (anticipatory responses, fast guesses) or longer than 3000 ms (delayed responses, lapses of attention) were not considered to be indicative of the cognitive processes involved in the current task and were excluded from the analysis as outliers (Connell, 2005, 2007). Also, reaction times 2.5 standard deviations above or below the mean in each condition (match vs. mismatch) for each participant were treated as outliers and excluded (De Koning, Wassenburg, Bos, van der Schoot, 2015; Ratcliff, 1993). This resulted in a removal of less than 5 % of the data. Accuracy for all remaining trials, excluding fillers, was high (M = .96, SD = 0.06). The high percentage of correct responses indicates that participants adequately understood the procedure. Importantly, for reaction time analyses only reaction times on correctly answered trials were used. The difference between the average reaction times on mismatch trials and match trials (RT_mismatch _− RT_match_) per child was calculated as an index of a child’s sensory richness of the mental text representation. We thereby follow the argumentation given by Zwaan and Pecher (2012): the larger the difference score, the more the child mentally simulated the object in the shape of how it was implied by the sentence (as a consequence of which a faster response on match trials could be given).

##### The situatedness of the mental representation

The situatedness of the mental text representation was measured by the standardized Test for Reading Comprehension of the Dutch National Institute for Educational Measurement (CITO; Feenstra et al., 2010). This test is a nationwide test to follow primary school student’s general reading comprehension skills in the Netherlands and is designed to be grade level appropriate. The test contains two modules (i.e., start module and follow up module) each consisting of a text and multiple choice questions. All children make the start module which contains 25 questions, after which the children are assigned (based on their scores on the start module) to either the follow up 1 module or the follow up 2 module. The follow up 1 module is designed for the less successful readers (which had a score between 0 and 12 on the start module), where the follow up 2 module is designed for the successful readers (which had a score between 13 and 25 on the start module). The follow up modules contains 25 questions in Grade 4 and 30 questions in Grade 5 and Grade 6.

Questions pertain to the word, sentence and text level, and, in addition to the text-based level of comprehension, tap into the situation-model level of comprehension (Kintsch, 1988). Due to CITO regulations only standardized, grade-normed proficiency scores were available for children’s performance on this test, which were obtained from the school teachers. The internal consistency coefficients of the Grade 4-, 5-, and 6- versions of the CITO Test for Reading Comprehension were good with Cronbach’s alpha’s of, respectively, .85, .90, and .88. Cronbach’s alpha’s were calculated using data from a previous norming study (Feenstra et al., 2010; Tomesen & Weekers, 2012).

### Mediators

#### Number of sensory words

For counting the number of sensory words in each written narrative, we used the approach described in Jampole et al. (1991). This scoring method consisted of counting all words in a narrative that refer to one of the following modalities: visual, auditory, tactile, kinesthetic, olfactory, organic, taste, emotional, and action words. This resulted in a ‘total number-of-sensory-words’-score for each child. Note that each score was the mean of the two raters’ scores. Since the children were allowed to spontaneously use as many sensory words as they liked, there was no maximum score on this variable. The maximum mean score reached in our sample was 48.00. The internal consistency between the two raters was excellent with a Cronbach’s alpha of .93.

#### Number of situational words

For counting the number of situational words in each written narrative, we drew upon prior work on reading comprehension research in general and the situational nature of mental text representations in particular (e.g., Zwaan et al., 1995). We took the five key situational dimensions (the ‘who’/‘what’, ‘where’, ‘when’, ‘how’ and ‘why’ of story passages) that lay the foundation for a story and counted, for each narrative, the words that referred to each of these dimensions. This resulted in a mean ‘total number-of-situational-words’-score (across the two raters) for each child. For the same reason as for the sensory words, there was no maximum score on this variable. The maximum mean score reached in our sample was 72.50. The internal consistency between the two raters was excellent with a Cronbach’s alpha of .93.

### Outcome variable

The originality/novelty of the story was measured by the ‘Novel Qualities’ subscale of the Carlson Analytic Scale for Measuring the Originality of Children’s Stories (Carlson, 1965). This subscale measures the originality of written narratives using 16 items: novelty of names, novelty of locale, unique punctuation and expressional devices, new words, novelty of ideas, novel devices, novel theme, quantitative thinking, new objects created, ingenuity in solving situations, recombination of ideas in unusual relationships, picturesque speech, humor, novelty of form, inclusion of readers, and unusual related thinking (Carlson, 1965). Following Jampole et al. (1991), each of the 16 items were scored on a four-point Likert scale, ranging from ‘absent’ (0) to ‘highly present’ (3). From these scores, a total score (0 - 48) was computed for each child reflecting his/her originality/novelty of writing. A mean score was computed across the two raters, demonstrating a good internal consistency (Cronbach’s alpha = .75).

### Procedure

As mentioned above, the standardized CITO Test for Reading Comprehension is part of the regular school curriculum assessment to follow children’s progress in reading comprehension. It takes a whole-class test taking approach and is administered by the classroom teacher in January–February each school year. The normalized scores were received from the school administration. One month later, the written narrative was administered, also by the regular classroom teacher, who followed a written protocol. The teacher explained to children that the assignment was to write a story that started with the sentence: ‘If I were invisible for one day…’. All children were provided with a protocoled definition of the word ‘invisible’ to ensure that the assignment was clear and the same for all children. Therefore, we may assume that all children understood what was expected from them. Except that there was a maximum writing time of 20 min, the assignment had no restrictions (e.g., there was no limitation of the number of words that could be used). The teacher warned children when they only had five minutes left.

After the narrative was written, children were individually tested on the SPVT in a silent classroom by a trained research assistant. We did not counterbalance the order of the writing task and the SPVT because the SPVT could influence children’s writing performance. Children sat behind a 15.6′ research laptop and were instructed to read each sentence at their own pace. Each trial started with a horizontally and vertically centered sentence on the computer screen, displayed in a black 24-point Courier New Bold font against a white background. Children pressed the spacebar when the sentence was understood, after which a 500 ms fixation cross appeared, followed by a picture. Participants indicated whether the pictured object was mentioned in the preceding sentence or not by pressing the keys marked by a green sticker (yes-response) and a red sticker (no-response). The task started with two practice trials to familiarize children with the task. Next, experimental and filler trials were presented in a random order. The SPVT took approximately 15 min to complete.

### Data analysis

Path analyses using MPlus Version 6 (Muthén & Muthén, 2010) were performed to examine which model fitted the data best: the direct model (with only direct pathways), the indirect model (with only indirect pathways), or the complete model (with both direct and indirect pathways). To assess model fit, a standard Maximum Likelihood method of estimating free parameters in path methods was used.

In evaluating the goodness of fit of the models, the Chi-square test statistic associated with a *p* value, the Confirmatory Fit Index (CFI), the Root-Mean-Square Error of Approximation (RMSEA), and Standardized Root-Mean-Square Residual (SRMR) values are reported. In this procedure, a non-significant Chi-square value, a CFI of more than 0.95, and a RMSEA and SRMR under 0.05 indicate a close fit. A CFI of more than 0.90, and a RMSEA and SRMR between 0.05 and 0.08 indicate an adequate fit. Finally, a significant Chi-square test, a CFI lower than 0.90, and a RMSEA and SRMR above 0.08 indicate poor model fit (Hu & Bentler, 1999; Kline, 2005).

To be able to investigate which model is best (in terms of model fit and model complexity), the Akaike’s information criterion (AIC) and the Bayesian information criterion (BIC) are reported. For both measures lower AIC and BIC values indicating a better model (Kline, 2005). Furthermore, in order to directly compare two nested models, we calculated the change in the Chi-square test statistic with correction for nested models using the Satorra-Bentler formula (Bryant & Satorra, 2012).

## Results

### Descriptive statistics

Table 2 shows the correlations, means, standard deviations, kurtosis, and skewness of the five variables in this study. All correlations reached significance, except for three: the correlation between sensory richness of the mental representation with the variables number of sensory words, number of situational words and situatedness of the mental representation.

**Table 2:** Correlations, means, standard deviations, kurtosis, and skewness for all variables

	1.	2.	3.	4.	5.	M (SD)	Kurtosis	Skewness
1. Sensory richness of MR	–	.09	.008	.07	.21*	62.93 (197.93)	0.47	0.20
2. Situatedness of MR		–	.30**	.21*	.27**	53.92 (21.58)	−0.82	0.18
3. Number of sensory words			–	.77**	.56**	12.92 (7.71)	3.10	1.49
4. Number of situational words				–	.43**	19.21 (10.53)	4.04	1.53
5. Originality/novelty of writing					–	4.97 (2.62)	0.14	0.63

*MR* mental representation

* *p* < .05; ** *p* < .01

### Comparing models

The direct and indirect model had poor fit indices on the Chi-square test and RMSEA. In addition, the direct model had a poor fit on SRMR and acceptable fit for CFI, where the indirect model had an acceptable fit for SRMR, and a close fit on CFI [direct model: χ^2^(5) = 15.469 *p* = .009; CFI = .95; RMSEA = .11; SRMR = .09, AIC = 6118.07, BIC = 6164.66; indirect model: χ^2^(3) = 11.84, *p* = .008; CFI = .96; RMSEA = .13; SRMR = .06, AIC = 6118.44, BIC = 6171.24]. The complete model, however, had a close fit on all fit indices [χ^2^(1) = .99, *p* = .32; CFI = 1.00; RMSEA = .00; SRMR = .02; AIC = 6111.59; BIC = 6170.60].

In order to test which model is the ‘best’ model, the complete model was compared to both the indirect and direct models. The results of these tests showed that adding terms to the direct and indirect model had surplus value, that is, the complete model is a significantly better model than both the direct model [Δχ^2^ (4, *N* = 165) = 17.20, *p* = .002] and the indirect model [Δχ^2^ (2, *N* = 165) = 9.24, *p* = .010]. This finding is supported by the finding that the AIC (AIC = 6111.59) of the complete model was lower than the AIC of either of the other two models (AIC = 6118.07 and AIC = 6118.44). In addition, the complete model had a lower BIC (BIC = 6170.60) compared to the indirect model (BIC = 6171.24), but not compared to the direct model (BIC = 6164.66). Taken together, we conclude that the complete model, in which the effects of the two text comprehension measures (sensory richness and situatedness of the mental representation) are partially mediated by their corresponding text production counterparts (respectively, number of sensory words and number of situational words), is the best model in terms of model fit and model complexity.

**Fig. 2:**
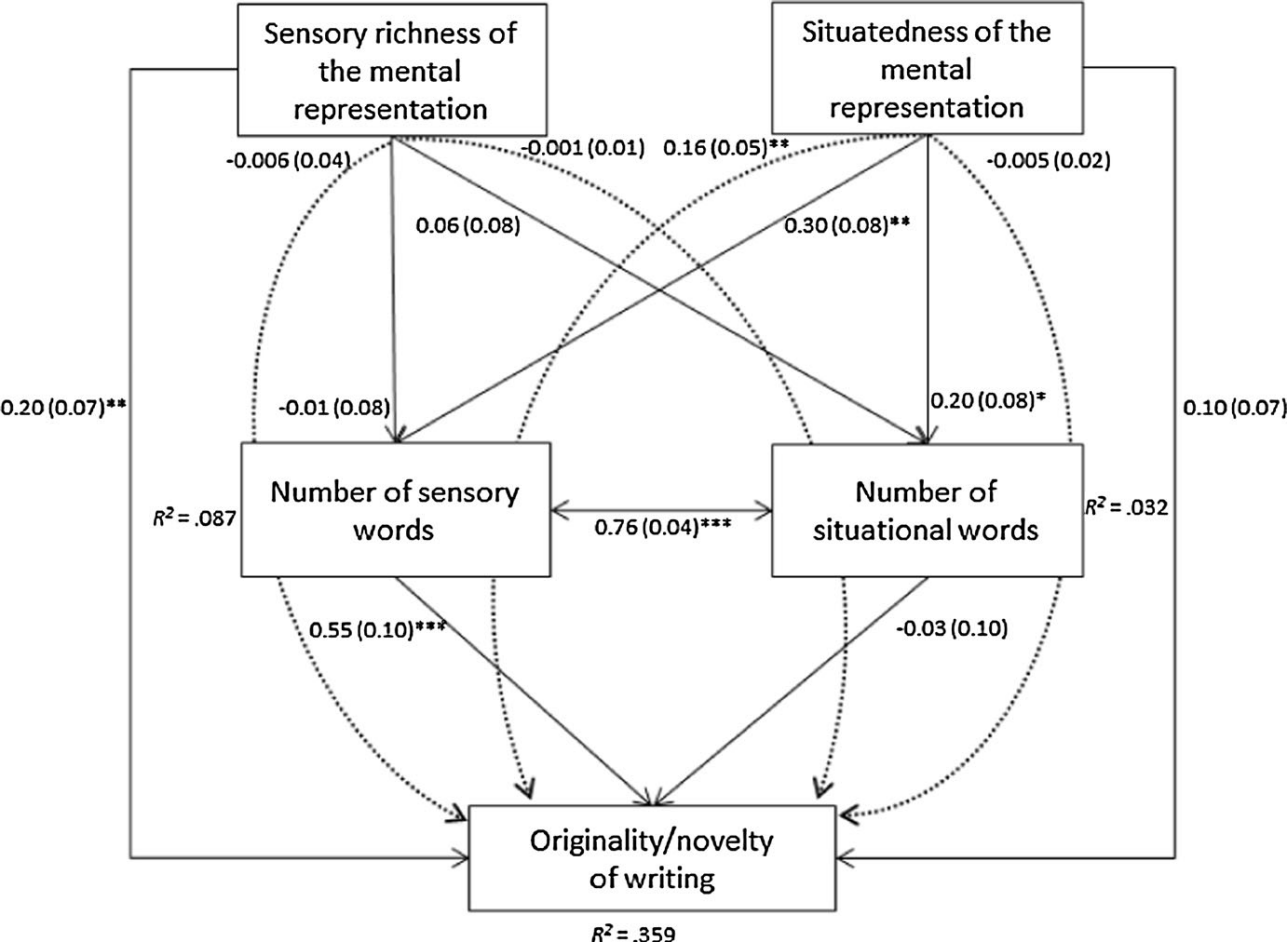
Complete model with standardized path coefficients (with standard errors in *brackets*) and percentages of explained variance (indicated by *R* ^2^). *Dashed lines* represent the indirect effects. *p < .05; **p < .01; *** *p* < .001

**Fig. 3:**
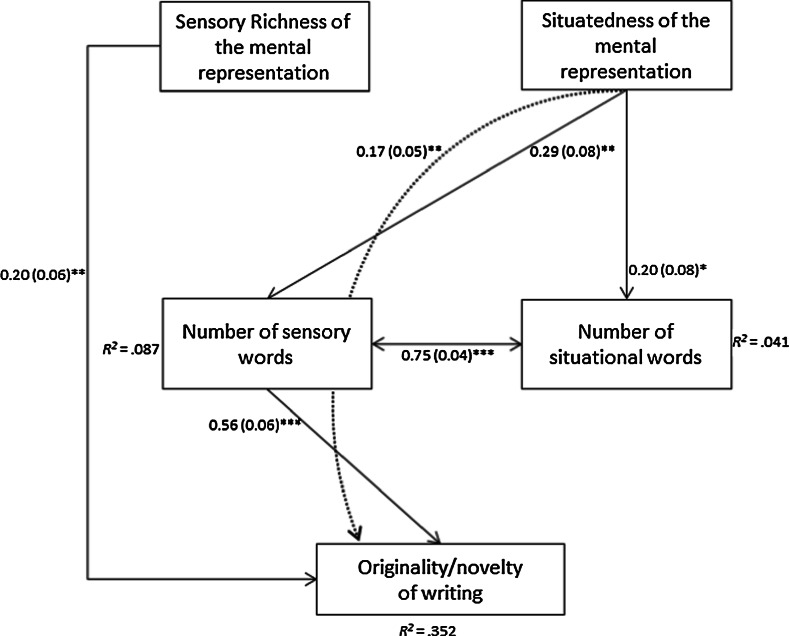
Final model with standardized path coefficients (with standard errors in *brackets*) and percentages of explained variance (indicated by *R* ^2^). *Dashed lines* represent the indirect effect. *p < .05; **p < .01; *** *p* < .001

Figure 2 shows the graphical representation of the complete model, including the standardized parameter estimates. As can be seen in this figure, the following pathways reached no significance: sensory richness of the mental representation towards the number of sensory words (*β* = −0.01, *p* = .890), situatedness of the mental representation towards originality/novelty (*β* = 0.10, *p* = .195), the number of situational words towards originality/novelty (*β* = −0.03, *p* = .807), and sensory richness of the mental representation towards the number of situational words (*β* = 0.06, *p* = .461). For the sake of parsimoniousness, we removed the non-significant pathways (see Fig. 3) and evaluated whether or not this model decreased in its fit indices. The goodness-of-fit indices of this final model, that is, the model in which only the statistically significant pathways were retained, indicated a close model fit [χ^2^(5, *N* = 165) = 4.46, *p* = .486, CFI = 1.00, RMSEA = .00, SRMR = .034, AIC = 6107.06, BIC = 6153.65]. Statistical comparison of this model to the complete model using the Satorra–Bentler formula did not reach significance [Δχ^2^ (4, *N* = 165) = 3.62, *p* = .46], that is, adding terms to the final model did not improve the model. In addition, both AIC and BIC were lower for the final model compared to the complete model (AIC = 6107.06, BIC = 6153.65). Consequently, we conclude that this final model is the better model, therefore we will discuss the results of this more parsimonious final path model below.

As can be seen in Fig. 3, the path analysis showed that 35.2 % of the variance in the creative narrative writing outcomes (i.e., originality/novelty) was explained by the sensory richness of the mental representation (*β* = 0.198, *p* = .002), the number of sensory words (*β* = 0.56, *p* < .001), and the indirect effect of situatedness of the mental representation on originality/novelty running via the number of sensory words (*β* = 0.165, *p* = .001). In addition, situatedness of the mental representation explained 4.1 % of the variance in the number of sensory words (*β* = 0.294, *p* < .001) and it explained 8.7 % of the variance in the number of situational words (*β* = 0.203, *p* = .013).

## Discussion

The present study investigated whether reading comprehension measures tapping the situatedness and sensory richness of mental representations also underlie children’s creative narrative writing outcomes. Besides determining their relative influences on creative writing, our objective was to see whether these measures can directly predict the originality/novelty of writing, or whether the relationship is mediated by their corresponding text production measures, respectively, the actual number of situational and sensory descriptions used in the stories.

The results of the path model analyses give an understanding of the elements involved in creative writing and how they are related to each other. Although sensory richness and situatedness of mental representations both influenced the creative writing outcomes of children, they followed their own distinctive pathway. Sensory richness was found to be directly related to the originality/novelty of their writing whereas the effect of situatedness was mediated by the number of sensory words in the texts they had produced. Together, the two variables explained roughly 35 % of the variance in creative writing, with both of them influencing the outcomes to a comparable extent as the estimation of the path coefficients was found to be 0.20 and 0.17, respectively.

The first and most obvious conclusion to be drawn from the former result is that the sensory richness of mental representations does not only play a role in reading comprehension (from text to mental representation; e.g., Zwaan & Pecher, 2012) but also in creative writing of a narrative (from mental representation to text). Remember that our reading comprehension measure of sensory richness reflects the extent to which readers mentally simulate the perceptual, motor, and emotional content of a narrative. Mental simulation is thought to be central to reading comprehension (Barsalou, 2008) and explains why the latter is generally considered as a process which involves (re-)creating multi-sensory experiences in one’s mind (De Koning & van der Schoot, 2013). Our results extend this idea by showing that the same can be said about creative writing of narratives. That is, while planning what to write about, writers ought to imagine the situations and events to be included in the story as vividly as possible, as if they are really experiencing them themselves (see also Bereiter & Scardamalia, 1987). At least, the path model results suggest that performing such mental simulations before and during writing helps writers to tap into their creative potential and hence write a narrative with originality/novelty.

What is of particular interest here is that the effect of mental simulations (i.e., the sensory richness of a mental representation) on creative writing outcome was direct in the sense that the assessed creativity did not lie in the number of sensory words used in the stories, but rather in the ability to use words in a way that evokes compellingly vivid, multi-sensory, images in the reader’s mind. Although sensory word use was found to have a (mediating) effect on children’s writing outcomes in the indirect pathway between the situatedness of mental representations and creativity of writing (as will be discussed later), the direct path from the sensory richness variable to creativity of writing indicates that mental simulation of the events one wants to write about (at least also) involves a mental process for which sensory language is not per se required. To understand this, it is useful to recognize that one-or-few-words creative expressions, such as the comparison made in the sentence ‘his house is like a zoo/prison/et cetera’, can be used to simultaneously evoke multiple sensory details (e.g., information about texture, temperature, taste, smell, size; see also Zimmerman & Hutchins, 2003). In a similar manner, a single word has the capacity to evoke a variety of mental images appealing to the reader’s senses depending on the context in which it was described (e.g., the word ‘bumpy’ used by an airline pilot warning his passengers for bumpy weather; De Koning et al., 2015; De Koning & van der Schoot, 2013). So, a child who writes about a ‘Hipagardocason’ (an animal which has combined features of a hippopotamus, giraffe, rhinoceros, dog, cat, and person; example taken from Carlson, 1965) obviously has a rich mental representation in which sensory details are creatively combined. Hence, this child would most probably get a high rating for creativity in spite of having used only one (pseudo)word to evoke this powerful multi-sensory image in the reader’s mind.

In contrast to its sensory richness, the situatedness of a writer’s mental representation influenced creative writing indirectly through the number of narrative descriptions of interest that children actually provided in the text. More in particular, the effect of this text comprehension measure on the originality/novelty of a story was mediated by the number of sensory words, not by the number of situational words. In light of the above considerations, attention should first be directed to the second step of this indirect pathway, as it shows that the amount of sensory language does have an impact on the originality/novelty of the text in which it occurs. This effect can be explained by assuming that sensory words contribute to the richness and vivid power of images created in the reader’s mind (Sadoski & Paivio, 2001). This notion is consistent with previous studies that addressed the relationship between sensory language and creative expression in language (e.g., Jampole et al., 1991, 1994). Above all, this relationship has been attributed to the fact that sensory words serve as an important tool in several types of figurative language. For example, similes (e.g. her breath is as fresh as a cool breeze), metaphors (e.g. love is a warm fire on a cold day) and personifications (e.g. the flowers danced in the gentle wind) are expressions of figurative language which often make use of sensory words. The close relation between sensory and figurative language is not difficult to understand, nor are their relations with creative originality. By definition, sensory words appeal to our senses, and as such, they can function to make one’s writing come to life when captured in figurative language, resulting in a more original and creative story (Stern and State 1988).

Perhaps the most striking feature of the path model concerns the source of sensory language as sensory word use seems to be rooted in the situatedness of a mental representation rather than in its sensory richness. Here, in an attempt to understand this finding it is important to consider the reading comprehension literature on embodied situation-models (De Koning & van der Schoot, 2013). In particular, two assumptions of embodied situation-model theory seem to be relevant. First, the situational dimensions of protagonist, time, space, intentionality, and causation are the cornerstones laying the foundation of a situation-model (Zwaan et al., 1995). Second, situation-models are implemented by the same sensorimotor neural representations formed while actually perceiving and acting out the described events (Barsalou, 1999, 2008). Against this background, the significance of our findings is that the use of sensory language in narrative writing seems to be related to the former feature of situation-models (indexed by the situatedness variable), more than to the latter (indexed by the sensory richness variable).

To clarify this further, it should be recognized that the situatedness of mental representation variable serves a double function in the path model, which tells us two things about writers who create a clear situational framework in their mind along the narrative dimensions of ‘who and what’, ‘where and when’, and ‘why and how’. First, they are inclined to use more situational words, that is, words which lay the foundations for a situation-based story structure. Second, they are more likely to bring their stories alive with sensory descriptions. Whereas the former finding was to be expected, the latter is somewhat surprising and deserves an explanation here. In our view, the path between situatedness of mental representations and sensory word use suggests that, in narrative writing, the dimensions of protagonist, time, space, intentionality and causality serve as the ‘mental clotheslines’ on which sensory descriptions can be hung (De Koning & van der Schoot, 2014). In other words, producing a situation-based mental representation of a story line before starting to write may incite children to include the senses in their writing and enrich the narrative they are working on with sensory details (and hence make it more original). Such enrichments likely differ between individuals due to their subjective nature and can be seen as an intra-personal enhancement of the constructed mental text representation (Long & Hiebert, 1985; Plum, 1982). On the contrary, as was discussed earlier, mentally simulating the events you have in mind before writing them down does not necessarily lead to more sensory language (albeit we have shown that mental simulation processes influence the originality/novelty of writing, only not through the use of sensory vocabulary).

### Educational implications

If the goal is to teach children to write creatively, the results of this study underscore the importance of representational skills as part of the methods used in (creative) writing instruction in primary schools. We have demonstrated that representational skills which are considered to be key in the field of reading comprehension are also relevant for children’s creative narrative writing outcomes. This has educational implications in the sense that teachers concerned with narrative writing instruction now can extend their repertoire of tools by incorporating aspects of situation-model and mental simulation theory, not only in reading comprehension lessons, but also in (creative) narrative writing lessons. For example, teachers should find appropriate ways to teach children how to create mental representations that involve situational plot structures (Zwaan et al., 1995) as well as sensorimotor simulations (Zwaan & Pecher, 2012). In doing so, situatedness and sensory richness should receive similar amounts of attention as they appear equally relevant for the creativity of writing (though in different ways).

Instructional approaches and strategies used in reading and writing lessons might fulfil similar or complementary roles. Moreover, aspects learned during reading lessons might be transferred to narrative writing instruction and vice versa. For example, while reading lessons might involve instruction on the importance of situatedness and sensory richness for narrative text comprehension, writing lessons may focus more on whether children are able to apply this knowledge when composing a narrative. Future research should examine whether and how this scenario actually can be implemented in school curriculum.

### Limitations

In considering the weight that should be given to the earlier interpretations and implications, we would like to discuss two limitations of the present study. First, we focused on creative expression in narrative, or story, writing. One must, therefore, be careful with generalizing the current results to other genres such as informational writing (e.g., reports), functional writing (e.g., formal letters), literary writing (e.g., poetry) or persuasive writing (e.g., stating an opinion). Different genres of writing not only have different purposes, structures, audiences, and conventions (Knapp & Watkins, 2005), but, more importantly here, also require and reward different kinds of creativity. Second, we did not differentiate the (level of) instruction to different grades. Since narrative writing outcomes likely develop over time, future research should consider grade-related differences and developmental trends in creative writing (including the role of mental model construction) and the potential need for grade-specific interventions. The results from the present study at least provide a starting point from which such endeavors could be further explored.

## Conclusions

Taken together, the findings of this study have two theoretical implications regarding the role of mental representations in creative writing. First, both situational and sensory word use in a creative narrative depend on the extent to which the mental representation which is generated prior to writing is situation-based, rather than to the extent to which it is sensory-rich. Second, the originality/novelty of a story is more related to the writer’s ‘sensory profile’ than to his ‘situational profile’, where ‘sensory profile’ refers to both the sensory aspects of his mental representation guiding the creative writing process as well as the sensory aspects of the actual story which has been produced (i.e., the amount of sensory language). With this study, we have extended previous research by showing that representational processes involved in narrative reading comprehension also underlie creative writing of narratives. In turn, this has implications for future writing research suggesting that its theoretical and applied orientation should be broadened to the various disciplines in reading comprehension research, in particular those related to mental representation and embodiment.
